# The effect of schizophrenia-associated CNVs on other psychiatric disorders

**DOI:** 10.1192/bjo.2021.665

**Published:** 2021-06-18

**Authors:** Lily Farakish

**Affiliations:** Divison of Psychological Medicine and Clinical Neuroscience, Cardiff University

## Abstract

**Aims:**

Schizophrenia is a highly heritable disorder, sharing genetic roots with other psychiatric disorders from both common and rare genetic variants. Copy number variants (CNVs) are one of the rare causes which increase the risk of a variety of psychiatric, medical and physical phenotypes. The role of schizophrenia-associated CNVs is becoming of increasingly scientific and clinical importance in the field of psychiatry, with new CNV-phenotype relationships opening perspectives for understanding the aetiology of psychiatric disorders. This paper aims to investigate whether 13 schizophrenia (SZ)-associated CNVs or any SZ-CNV-carrier status increase the risk for 9 psychiatric phenotypes, reduce levels of happiness, change duration of sleep, and increase the index of multiple deprivation.

**Method:**

The study includes 421,268 participants of British or Irish descent (aged 40–69 years), containing 418,036 controls and 3232 schizophrenia-associated CNV carriers. The data are secondary from the UK Biobank, an online resource containing data on array-genotyped participants with their specific phenotypic information. Prior to analysis, CNV selection led to the exclusion of any CNV with less than 5 hits in the UK Biobank population. Incidence of each phenotype was based on self-reported diagnoses, questionnaires or hospital ICD-10 diagnoses, with a minimum of 500 cases. Both binary logistic and linear regression were used to assess the incidence of these phenotypes in relation to the CNVs, adjusted for age, sex, and ethnicity as potential cofounders.

**Result:**

Overall, 12/13 CNVs were nominally associated with at least one phenotype, including 114/168 possible associations and 54 undetectable associations as not every CNV carrier displayed one of the chosen phenotypes. 41 associations were statistically significant (p < 0.05) and 13 survived Bonferroni Correction (p < 2.98 × 10-4). All significant associations met the expected change except 15q11.2 deletion and any CNV carrier status which showed a decrease in likelihood of addiction.

**Conclusion:**

These findings suggest schizophrenia-associated CNV can affect range of psychiatric phenotypes. By building on existing reports, understanding the widespread effects of CNVs in the aetiology and pathogenicty of psychiatric disorders may overtime aid in strengthening our search for more targetted, effective treatments.

Many thanks to Professor George Kirov for supervising and supporting this project.

